# Spinal mobility in radiographic axial spondyloarthritis: criterion concurrent validity of classic and novel measurements

**DOI:** 10.1186/s12891-021-04352-z

**Published:** 2021-05-21

**Authors:** John Charles Snow, Kyle Simpson, Proton Rahman, Samuel Howarth, Diana De Carvalho

**Affiliations:** 1 Division of Community Health and Humanities, Faculty of Medicine, Memorial University of Newfoundland, 300 Prince Philip Dr, St. John’s, NL A1B 3V6 Canada; 2 Private Practice, Kitchener, ON N2M 1Y5 Canada; 3 Discipline of Medicine, Division of Rheumatology, Faculty of Medicine, Memorial University of Newfoundland, St. John’s, NL A1B 3V6 Canada; 4 Division of Research and Innovation, Canadian Memorial Chiropractic College, Toronto, ON M2H 3J1 Canada

**Keywords:** Range of motion articular, Spine, Physical examination, Radiography, Spondylarthritis, Accelerometry

## Abstract

**Background:**

Limitations in spinal mobility are a characteristic feature of Axial Spondyloarthritis. Current clinical measurements of spinal mobility have shown low criterion-concurrent validity. This study sought to evaluate criterion-concurrent validity for a clinically feasible measurement method of measuring spine mobility using tri-axial accelerometers.

**Methods:**

Fifteen radiographic-Spondyloarthritis patients were recruited for this study. Two postural reference radiographs, followed by three trials in forward, left and right lateral bending were taken. For all trials, three measurements were collected: tape (Original Schober’s, Modified Schober’s, Modified-Modified Schober’s, Lateral Spinal Flexion Test and Domjan Test), followed immediately by synchronized radiograph and accelerometer measurements at end range of forward and bilateral lateral flexion. The criterion-concurrent validity of all measurement methods was compared to the radiographic measures using Pearson’s correlation coefficients. A Bland-Altman analysis was conducted to assess agreement.

**Results:**

In forward bending, the accelerometer method (r = 0.590, *p* = 0.010) had a stronger correlation to the radiographic measures than all tape measures. In lateral bending, the Lateral Spinal Flexion tape measure (r = 0.743, *p* = 0.001) correlated stronger than the accelerometer method (r = 0.556, *p* = 0.016). The Domjan test of bilateral bending (r = 0.708, *p* = 0.002) had a stronger correlation to the radiographic measure than the accelerometer method.

**Conclusions:**

Accelerometer measures demonstrated superior criterion-concurrent validity compared to current tape measures of spinal mobility in forward bending. While a moderate correlation exists between accelerometer and radiographs in lateral bending, the Lateral Spinal Flexion Test and Domjan Test were found to have the best criterion-concurrent validity of all tests examined in this study.

## Background

Axial spondyloarthritis (AxSpA) is a chronic and progressive inflammatory disease affecting the axial skeleton with a prevalence between 0.2 and 1.4% in North America [1, 2]. Disease progression and treatment response are monitored by objective indicators of spine mobility in forward and lateral bending using clinical tape measures [3]. Specifically, spine mobility of AxSpA patients is assessed using clinical tests in the sagittal (Original Schober’s Test (OST) [4], Modified Schober’s Test (MST) [5], Modified-modified Schober’s Test (MMST) [6]) and frontal planes (Lateral Spine Flexion Test (LSFT) [7], Finger-Fibula distance [8], Domjan test (DT) [9]). Multiple previous studies have reported poor criterion-concurrent validity of using a tape measure to assess spine mobility, compared to the gold standard of spine angles and range of motion (RoM) obtained from radiographs [4, 6, 10, 11]. Combining the risks associated with repeated exposure to ionizing radiation for obtaining radiographs and the aforementioned lack of criterion-concurrent validity for current clinical measures of spine mobility necessitates a search for clinically viable alternatives for evaluating spine mobility in AxSpA patients.

Inclinometry is a reliable and valid way of measuring spine angles and may be beneficial for monitoring angles in this clinical population. Devices that incorporate strain gauges and/or accelerometers, such as the Epionics SPINE system and The Spinal Mouse have been shown to have excellent inter and intra-rater reliability for the measurement of spinal curvature and the lumbar lordosis angle [12, 13]. Tri-axial accelerometers can be used as inclinometers and have the advantage of using 3 axes to accurately calculate angles. Lumbar spine angles measured with inclinometers or accelerometers have previously reported excellent accuracy (≤ 1° RMS error) [14] and reliability (ICC = 0.964 and r = 0.91 respectively) [15, 16]. Furthermore, these sensors are routinely used in biomechanics studies to quantify spine angles when tasks are relatively static (e.g. office and automotive seating studies) and line of sight issues preclude the use of optoelectrical motion capture systems [17, 18]. Since the relative angle between two sensors placed at the top and bottom of a spine curve mathematically parallels the measure taken at the vertebral bodies on plain film radiographs, it is logical that these measures may have a higher criterion-concurrent validity than tape measures that curve along the back at the static end RoM.

To date the true criterion-concurrent validity of these sensors compared to radiographic measures of spinal mobility has not been tested. Therefore, this study evaluated spine RoM measured by tri-axial accelerometers compared to both current clinical tests and radiography in radiographic-AxSpA patients.

## Methods

### Hypothesis

Tri-axial accelerometers would provide a stronger Pearson (r) correlation coefficient than traditional tape measures when compared to radiographic gold standard measure of spinal mobility.

### Participants

Recruitment and data collection occurred from January 2018 to March 2018. Individuals were recruited from disease-specific interest groups and rheumatology practices. Adults with a confirmed diagnosis of radiographic-AxSpA were included [19]. Individuals occupationally exposed to radiation and/or women with the chance of being pregnant were excluded. The Newfoundland and Labrador Health Research Ethics Authority granted ethics approval prior to the start of this study (#2017.057). The Canadian Memorial Chiropractic College also granted ethics approval prior to the start of this study (#172014)*.* Participants gave informed written consent prior to beginning the data collection.

### Disease indices

Each participant completed the Bath Ankylosing Spondylitis Disease Activity Index (BASDAI).

### Landmarks and reference points

**Fig. 1:**
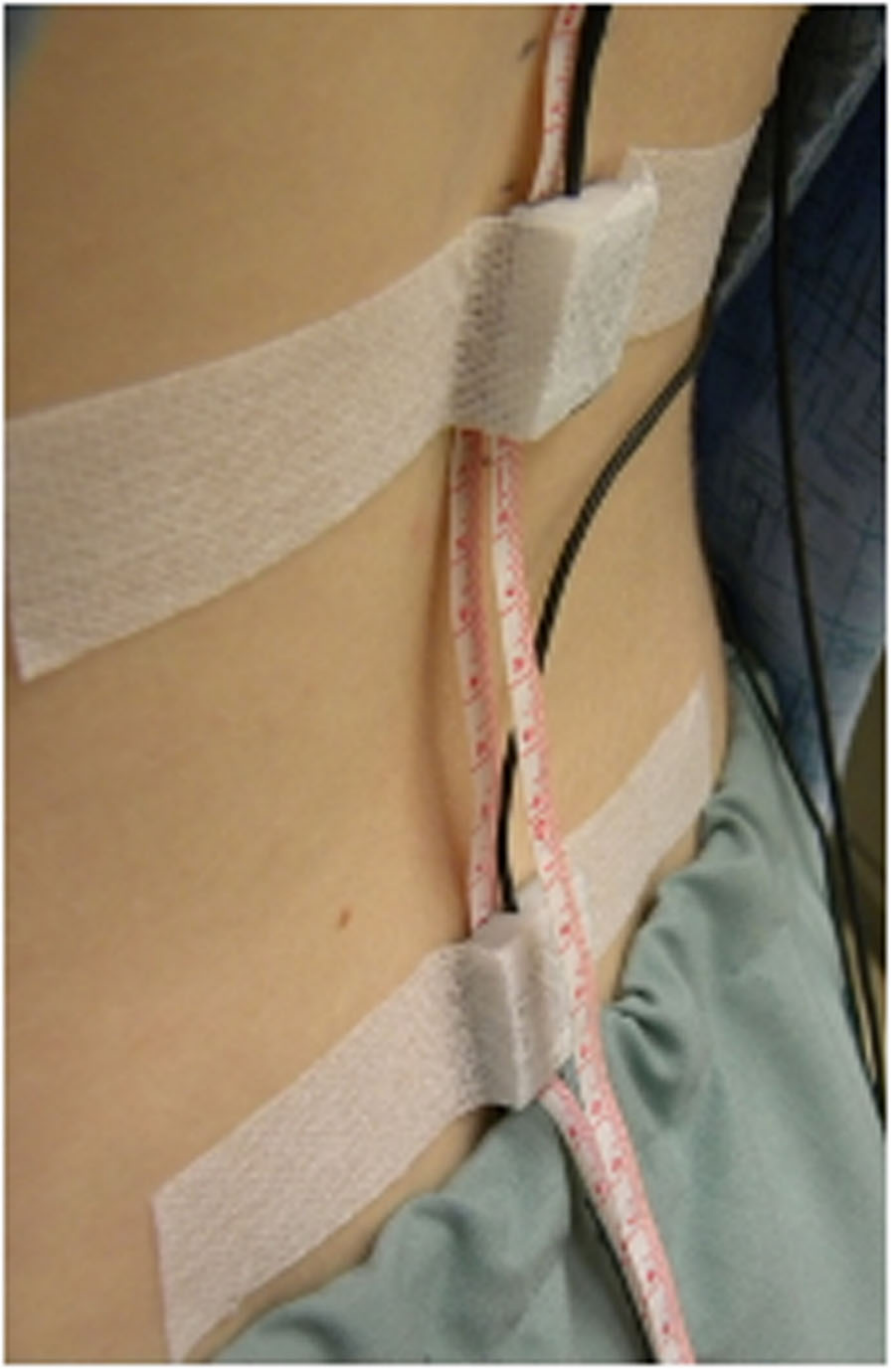
Visual representation of two tape measures lying underneath the L1 and S1 accelerometers

Lumbar spinous processes (L1-L5) were identified by manual palpation using anatomical landmarks. These locations were marked with a washable pen for sensor placement at L1 and the sacrum. A horizontal line was drawn inferior to the spinous process of L5, approximating the lumbosacral junction (LSJ) [20]. With the participant still standing, separate horizontal lines were drawn 5 cm below, and 10 and 15 cm above the line representing the LSJ (Fig. 1). Different combinations of these 4 lines were used to determine measurements for the OST, MST and MMST.

### Instrumentation

#### Measuring Tapes

Two standard clinical measuring tapes were affixed to the skin overlying the midline of the participant’s low back so they could slide under the accelerometers throughout forward bending (Fig. 1). One measuring tape was positioned where 0 cm coincided with the marking made 15 cm above the LSJ. The second measuring tape was positioned such that 0 cm coincided with the marking made 10 cm above the LSJ. This method ensured measurements were taken from the same place while minimizing the time to obtain the OST, MST and MMST measures.

#### Tri-axial accelerometers

Two tri-axial accelerometers (ADXL335, Analog Devices, Norwood, MA, USA) were calibrated to gravity prior to the start of data collection and fixed to the skin overlying the spinous processes of L1 and S2 in the +y down orientation [21]. Accelerometer fixation was achieved using double-sided tape on the edges of the sensor so that the tape measures could freely pass underneath the accelerometer at L1 (Fig. 1). Fabric tape was used over each accelerometer to mitigate sensor movement relative to the skin during movement. Accelerometer data were digitally sampled at 256 Hz using a ± 10 V range on a 16-bit analog to digital conversion board using custom Matlab software (The Mathworks Inc., Natick, MA, USA).

#### Radiography

Participants were fitted with thyroid and gonadal shielding. Technique factors were set based on torso thickness, measured in both the sagittal and frontal planes. For all views, collimation was set to include the vertebral bodies of T12 and S3. All films were taken with a diagnostic x-ray high voltage generator machine (HFQ-12050P, Toshiba, Bennett X-ray Technologies Inc., Copiague, NY, USA) by an experienced (42 years) Registered Radiologic Technologist with a 36 by 43-cm film size using 400 speed screen digital cassettes. Radiographic exposures were synchronized to accelerometer data using a custom-built switch.

### Experimental protocol

**Fig. 2:**
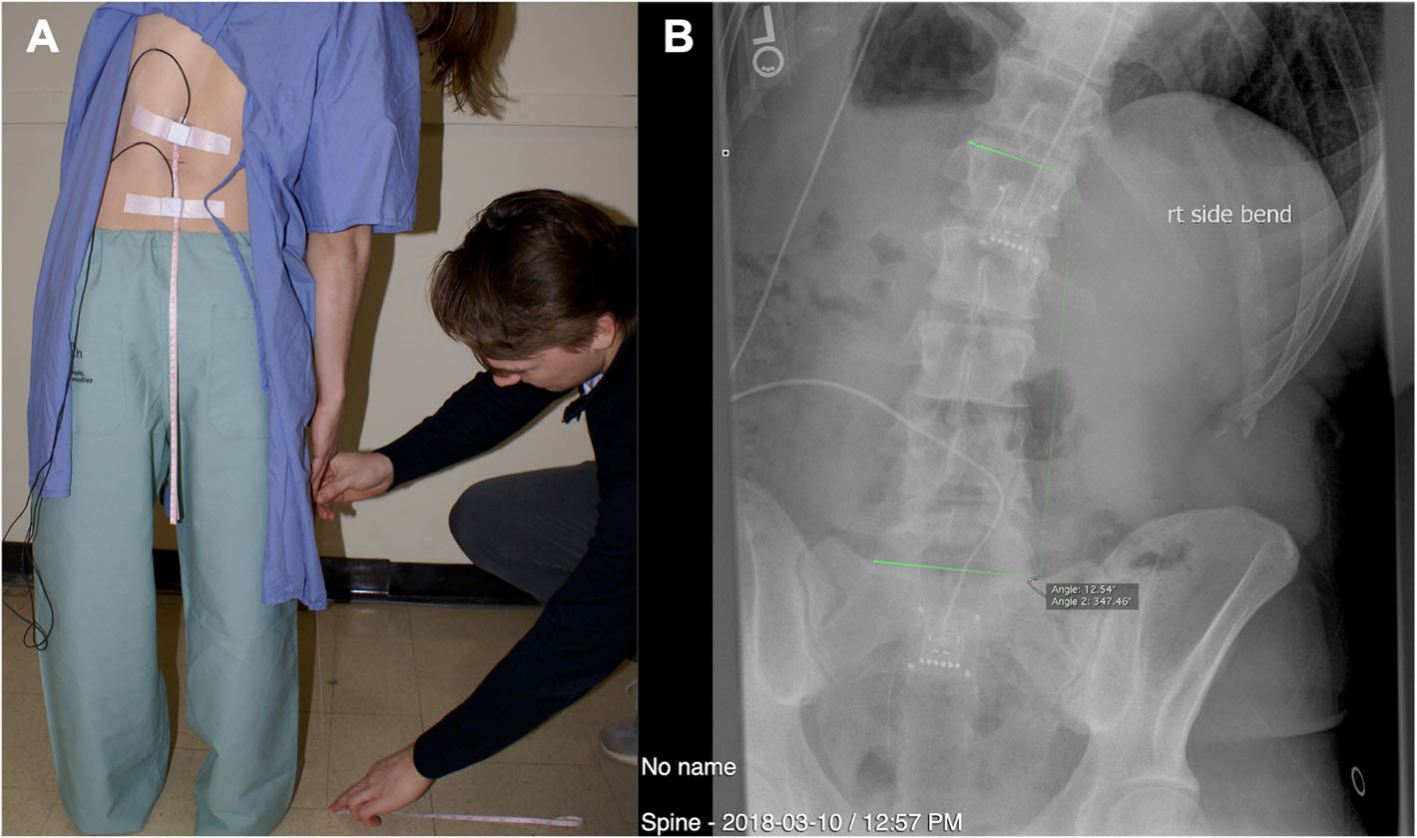
**a** Illustration of participant positioning for right side bend measures (fingertip to floor distance) and **b** representative radiograph of participant in right side bend with associate measures

Following instrumentation, separate lateral and posterior-anterior radiographs were taken, in a random order, with the participant standing upright. Each participant then completed three RoM trials; one in each of forward flexion, right lateral and left lateral bending in a random order. A total of 5 lumbar radiographs were obtained for each participant. All trials were performed in standing with feet shoulder width apart. For forward flexion, the participant was instructed to maximally bend forward, reaching fingertips to the floor keeping knees straight. For lateral bend, the participant was instructed to maximally bend to one side, keeping their trunk oriented in the frontal plane, with arms freely hanging by their sides*.* Each film was taken on suspended expiration to minimize superimposition of the diaphragm over the upper lumbar vertebral bodies. The participant held each end-range position for approximately 7 s to obtain both tape measure and radiograph/accelerometer data*.* A single investigator (JCS) performed all measuring tape measurements to the nearest millimeter. Figure 2 illustrates a representative right lateral bend trial with a corresponding radiograph. The first film of the series was screened by the technician and researcher to ensure positioning of the lead shielding did not obscure the superior endplate of S1 or the inferior endplate of L1.

### Spine mobility measures

#### Forward flexion measures of spine mobility

The OST, MST and MMST measures were made concurrently from the measuring tapes affixed to the skin overlying the participant’s back. The OST was made between the lines marked at the LSJ and 10 cm above the LSJ. The MST was made between the lines marked 5 cm below and 10 cm above the LSJ. Finally, the MMST was made between the lines marked at the LSJ and 15 cm above the LSJ. Differences between each of these measurements and the respective starting distance between relevant lines were used as the final measurements for the OST, MST and MMST.

#### Lateral bend measures of spine mobility

The LSFT was taken in standing with the participants’ arms at their sides. During each lateral bending trial, the distance between the tip of the middle finger on the ipsilateral side to the movement and the floor was measured once the participant had reached their end RoM. The difference between fingertip to floor in standing and at maximum flexion was used for both the left and right sides. The LSFT measure was recorded as the average of the difference measurements that were obtained from the left and right lateral bending trials. For the DT, two lines were marked on the side of the participant’s right leg coinciding with the position of the middle fingertip of the right hand at end RoM in both left and right lateral bend. The distance between these lines represents the DT measure.

#### Accelerometer measure

Post-collection processing of accelerometers data was completed with custom Matlab software [22]. These data were digitally filtered using a dual-pass 2nd order Butterworth filter with a cutoff frequency of 1 Hz and converted to acceleration using calibration factors. Sagittal and frontal plane inclination angles were determined from accelerations using the inverse tangent function for the duration corresponding to the radiographic exposure for all trials. Relative inclinations between upright standing and the end RoM in each trial were calculated as the difference between the top and bottom sensor. Lumbar spine angles from the two lateral flexion trials were averaged to represent the accelerometer measure of lateral bend mobility.

#### Radiographic measure

**Fig. 3:**
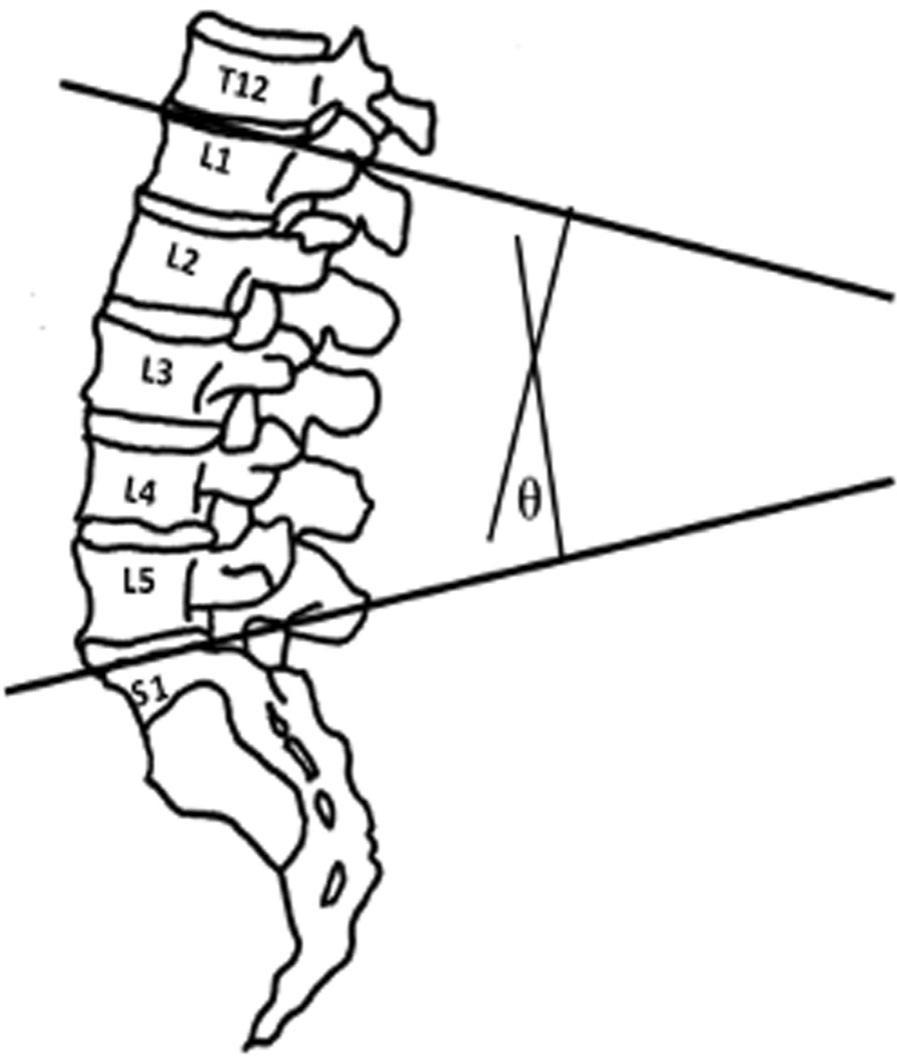
Method of calculating the lumbar spine angle from posteroanterior plain radiograph. Horizontal lines are drawn parallel and through the superior endplate of L1 and the superior endplate of S1. Perpendicular lines are drawn from the two original lines and the large angle at their intersection is measured

**Fig. 4:**
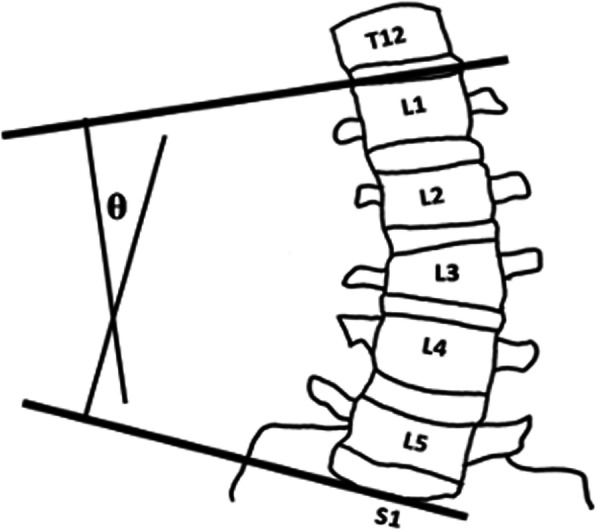
The radiographic method of calculating the lumbar spine angle from a lateral projection radiograph film. Horizontal lines are drawn parallel and through the superior endplate of L1 and the superior endplate of S1. Perpendicular lines are drawn from the two original lines and the large angle at their intersection is measured

Digital films obtained in this study were blinded and randomly presented to an investigator (JCS) for measurement of lumbar curvature angles in sagittal and frontal planes (Horos v2.4.0, Pixmeo SARL, Geneva, Switzerland). Two lines were drawn corresponding to the superior endplates of L1 and S1. Perpendicular lines were projected from each of the endplate lines until they intersected. The larger of the two acute angles at the intersection represented the lumbar curvature angle (Figs. 3 & 4). For lateral radiographs, this angle represented the lumbar lordosis angle. The change in lumbar curvature angle between upright standing and end RoM represented the lumbar spine angle for forward flexion, right lateral bend and left lateral bend. Lumbar spine angles measured from the right and left lateral flexion trials were averaged to represent the radiograph-derived measure of lateral bend mobility.

### Statistical analysis

Statistical analyses were completed with SPSS (SPSS Statistics 23, IBM Software, Armonk, NY, USA). Pearson correlations and their 95th percentile confidence intervals were calculated between the radiographic angles and the corresponding tape or accelerometer measures. A Bland-Altman analysis evaluated the presence of systematic differences between the radiographic and clinical tape or accelerometer measures respectively. A *p*-value of less than 0.05 and absolute value of any correlation coefficient exceeding 0.20 were considered significant.

Intra-rater reliability for radiographic measures of spine mobility was assessed by having the same investigator (JCS) perform repeated measurements of the radiographic angles from two sets of films (frontal and lateral) for all fifteen participants. These measures were taken at the same time of day on three consecutive days. Inter-rater reliability was assessed by having a second investigator (KS) determine the radiographic angles from the same set of images. Intra-class correlation coefficients (ICC_2,1_) were used to determine the inter-rater and intra-rater reliabilities of radiographic measures.

## Results

### Participant characteristics

Fifteen individuals (9 females, 6 males) participated in this study (Table 1).

**Table 1:** Summary of participant characteristics

Characteristics	Mean ± SD
Age (years)	45.9 ± 15.1
Height (cm)	167.1 ± 9.4
Weight (kg)	85.4 ± 17.8
Time since Diagnosis (years)	11.7 ± 9.4
BASDAI Score	5.5 ± 2.5
BASDAI Score in disease inactive population (BASDAI < 4)	2.3 ± 0.7
BASDAI Score in disease active population (BASDAI > 4)	6.7 ± 1.7

### Sagittal plane spine mobility

Correlations between spine mobility measures obtained using tape and radiographic measures of spine mobility were not statistically significant (Table 2). The accelerometer measure of spine mobility was moderately correlated (r = 0.59) with the radiographic lumbar spine angle (*p* = 0.010). The Bland-Altman analysis did not reveal any systematic difference between the instruments (t = 0.717, *p* = 0.486).

**Table 2:** Summary correlations for spinal mobility tests in sagittal plane bending

Measurement Tests Compared	Pearson Correlation Coefficient (r)	***P***-value (***P***)	95% CI
OST vs. Radiograph	0.195	0.243	[−0.318, 0.726]
MST vs. Radiograph	0.295	0.143	[−0.259, 0.748]
MMST vs. Radiograph	0.414	0.063	[−0.095, 0.731]
Accelerometer vs. Radiograph	0.590	0.010^a^	[0.235, 0.907]

^a^ indicates statistical significance at an alpha level of 0.05. *OST* Original Schober’s test. *MST* Modified Schober’s test. *MMST* Modified-modified Schober’s test

### Frontal plane spinal mobility

Strong correlations were observed between the radiographic measure of spine mobility in the frontal plane and measurements obtained from the LSFT (r = 0.74, *p* = 0.001) and DT (r = 0.71, *p* = 0.002). Correlation between the radiographic measure of spine mobility and accelerometer lumbar spine angle was moderate (r = 0.56, *p* = 0.016) (Table 3). The Bland-Altman analysis suggested that accelerometers systematically underestimated the radiographic angle by 3.6 ± 5.5 degrees in lateral bending (t = 2.544, *p* = 0.023).

**Table 3:** Summary correlations for spinal mobility tests in lateral bending

Measurement Tests Compared	Pearson Correlation Coefficient (r)	***p***-value (p)	95% CI
LSFT vs. Radiograph	0.743	0.001^a^	[0.456, 0.937]
Domjan vs. Radiograph	0.708	0.002^a^	[0.511, 0.867]
Accelerometer vs. Radiograph	0.556	0.016^a^	[0.113, 0.827]

^a^ indicates statistical significance at an alpha level of 0.05. *LSFT* Lateral spinal flexion test

### Intra-rater and inter-rater reliability of radiographic measures

Strong intra-rater (ICC_2,1_ = 0.98) and inter-rater (ICC_2,1_ = 0.97) reliability was observed for radiographic measures.

## Discussion

This investigation supports using accelerometers as a possible replacement for existing clinical measures of sagittal, but not lateral, spine mobility in patients with radiographic-AxSpA. Our dataset also showed, for the first time, that lateral clinical tape measures of spine mobility have excellent criterion-concurrent validity.

The OST and its derivatives (i.e., MST and MMST) are the current clinical measures for diagnosing and monitoring sagittal plane spine mobility in patients with AxSpA. Consistent with previously published work, this study shows weak to moderate correlations between these measures of sagittal plane spine mobility and the radiographic gold standard [4]. One possible explanation for these poor correlations is that the measures of sagittal plane spine mobility (OST, MST and MMST) are affected by stretching of the skin. For example, large systematic differences at end ranges of spinal flexion have been reported when using the current clinical tape measures [5]. At larger ranges of flexion the skin begins to slide across the underlying tissues rather than continuing to stretch, thereby causing disproportional changes to the Schober’s measurement [5]. Accelerometers present an interesting alternative for measuring sagittal plane spine mobility partly because their measurement is less influenced by skin stretching. Correlation between measures of sagittal plane spine mobility derived using accelerometers and the radiographic gold standard, reported herein, is an improvement over current clinical measures; however, the relationship using accelerometer-derived measures of sagittal plane spine mobility only explained 35% of the variance in spine mobility, which was consistent for the frontal plane. Further, accelerometer-derived measures of frontal plane spine mobility systematically underestimated lateral bend angles from the radiographs and demonstrated a moderate correlation to the radiographic gold standard that was lower than either the LSFT or DT. The precise reason for this systematic difference is unknown; however, modeling techniques could potentially be employed to correct for these systematic differences. Future studies could include a larger dataset to develop and test such models.

Despite the fact that clinical measures of frontal plane spine mobility (LSFT and DT) are commonly used to assess radiographic-AxSpA patients [23], the criterion-concurrent validity for these measures had not been previously established. Strong correlations observed between each of the clinical measures to the radiographic gold standard in this study suggest strong criterion-concurrent validity for the LSFT and DT. This is likely due to a combination of reduced impact of skin stretching and reduced variability in the measure since an average of measurements from left and right lateral bending is used to represent the clinical measurement. Taking the average from repeated measurements of spine mobility during forward bending could be considered as a potential way to improve the reliability and criterion-concurrent validity of the sagittal tape measures. Future work could examine this in more detail. This study’s findings support using lateral measures of spine mobility (not sagittal) for monitoring disease progression.

There are several limitations to this study. Although we used a broad recruitment strategy, this study was limited to a 3-month collection phase resulting in a small sample size. While the small sample limited the statistical power of the comparison between accelerometer and radiographs, it was great enough to determine the validity of the Domjan test, and to determine that it would be worth pursuing future research to develop accelerometers as tools to assist with the diagnosis and monitoring of spinal mobility impairments. A second limitation is that participants included in this study had advanced stages of the disease. There is a chance that correlations between measures might have been different in a population with greater range of spinal mobility. Future studies should include a larger patient cohort, with a range of clinical stage of disease, and a follow-up period to validate these findings in a more generalizable and clinically relevant way. Finally, there were limits to the instrumentation we used in this study. The underestimation of lateral bend spine angles as identified by the Bland Altman Analysis suggests a systematic difference in the measure obtained from the accelerometers. As previously mentioned, it is possible that employing modelling techniques to correct for these differences could improve correlations. Also, the tri-axial accelerometers are not sensitive to axial rotations within the transverse plane, thus these ranges were not tested in this study. There are other sensors that can measure twist in addition to sagittal and lateral bending such as inertial motion sensors. Thus, possible directions for future research includes exploring different sensors in combination with modelling techniques.

## Conclusions

Findings from this study reaffirm the lack of criterion-concurrent validity of the OST and MST tape measures previously reported in the literature. Results from the LSFT and DT suggest that these are valid methods for monitoring lateral spinal mobility limitations and are likely superior to the current sagittal measures. Accelerometers appear to have better criterion-concurrent validity for sagittal, but not frontal, plane measures of spinal mobility. Given the need to limit exposure to ionizing radiation through repeated use of radiographs in the AxSpA population, future work should focus on continuing to improve clinical measures of spine mobility. Improved clinical measures of spine mobility will provide clinicians with greater certainty when evaluating an individual’s disease progression and response to treatment, thereby improving clinical management.

## Data Availability

The full dataset will be made available upon request.
